# Retraction: QiShenYiQi Attenuates Renal Interstitial Fibrosis by Blocking the Activation of β-Catenin

**DOI:** 10.1371/journal.pone.0297842

**Published:** 2024-01-23

**Authors:** Zhanmei Zhou, Zheng Hu, Mei Li, Fengxin Zhu, Hao Zhang, Jing Nie, Jun Ai

The corresponding author requested retraction of this article [1] due to concerns about the published results. Specifically,

- The following immunohistochemical staining results appear similar:
  - The Sham fibronectin panels in Figure 1D and Figure 2D.
  - The vehicle fibronectin panel in Figure 1D and the vehicle collagen I panel in Figure 2D.
- The following western blot results appear similar:
  - The fibronectin panel in Figure 2E* of [1], the collagen I panel in Figure 4B* of [1], and lanes 1–6 of the α-SMA panel in Figure 2F of [2].
  - Lanes 1–4 of the collagen I panel in Figure 3B* of [1] and the IP:Smad3 IB:TβRI panel in Figure 6B of [2].
  - The α-SMA panel in Figure 4B* of [1] and the α-SMA panel in Figure 5E of [2].
  - The fibronectin panel in Figure 5B* of [1] and the fibronectin panel in Figure 5F of [2].

The corresponding author stated that errors in figure preparation occurred due to the studies being carried out around the same time. The original data underlying this study [1] are no longer available. In the absence of the original data, the above issues cannot be resolved.

In light of the unresolved concerns that call into question the validity and reliability of the reported data, the authors retract this article.

The panels marked with an * above report material from [2] which are not offered under a CC-BY license and are therefore excluded from this article’s [1] license. At the time of retraction, the article [1] was republished to note this exclusion in the relevant figure legends and the article’s copyright statement. For information about the copyright that applies to the excluded panels, please refer to [2].
